# Differential miRNA expression profile and proteome in plasma exosomes from patients with paroxysmal nocturnal hemoglobinuria

**DOI:** 10.1038/s41598-019-40453-5

**Published:** 2019-03-05

**Authors:** Raúl Teruel-Montoya, Ginés Luengo-Gil, Fernando Vallejo, José Enrique Yuste, Nataliya Bohdan, Nuria García-Barberá, Salvador Espín, Constantino Martínez, Juan Carlos Espín, Vicente Vicente, Irene Martínez-Martínez

**Affiliations:** 1 Servicio de Hematología y Oncología Médica, Hospital Universitario Morales Meseguer, Centro Regional de Hemodonación, Universidad de Murcia, IMIB-Arrixaca, Murcia, Spain; 2 Grupo de Investigación CB15/00055, Centro de Investigación Biomédica en Red de Enfermedades Raras (CIBERER), Instituto de Salud Carlos III (ISCIII), Madrid, Spain; 3 Servicio de Metabolómica, CEBAS-CSIC, 30100 Campus de Espinardo, Murcia, Spain; 4 Laboratory of Food & Health, Group of Quality, Safety and Bioactivity of Plant Foods, CEBAS-CSIC, 30100 Campus de Espinardo, Murcia, Spain

## Abstract

Paroxysmal Nocturnal Hemoglobinuria (PNH) is a clonal disease of blood cells caused by the lack of glycosyl phosphatidyl inositol anchored proteins bound to the cell membrane. In consequence, erythrocytes lead to intravascular hemolysis upon complement activation, which promotes high risk of thrombosis, intravascular hemolytic anemia, and bone marrow failure in patients. The mechanisms of thrombosis in PNH are still poorly understood. Treatment with eculizumab reduces intravascular hemolysis and thrombotic risk, but not in all cases. Exosomes are extracellular vesicles released by cells and whose secretion is closely related to the inflammatory status. They participate in cell communication by activating signaling pathways and transferring genetic material and proteins to host cells. In consequence, exosomes may serve as surrogate biomarkers for the prognosis and/or diagnosis of a disease. Isolation of exosomes was carried out from healthy controls and from three groups of PNH patients, i.e. i) with no eculizumab treatment; ii) under treatment with eculizumab that have not suffered thrombosis; and iii) under treatment with eculizumab but that have suffered thrombosis. The miRNAome and proteome was analyzed using plasma focus miRNAs PCR panel and LC-MS analysis respectively. We found differential expression of miRNAs miR-148b-3p, miR-423-3p, miR29b-3p, miR15b-5p, let-7e-5p, miR126-3p, miR-125b-5p and miR-376c-3p as well as hemoglobin, haptoglobin, protein S and C4-binding protein in healthy controls *vs* PNH patients. Our results warrant further research and provide new information on the content of exosomes that could play a role in the hypercoagulable state in this disease.

## Introduction

Paroxysmal nocturnal hemoglobinuria (PNH) is a rare, systemic disease associated with the deficiency of certain proteins in the erythrocyte membrane^1^. It is caused by a somatic mutation of the phosphatidylinositol glycan A gene (PIG-A) in the stem cells of the bone marrow, resulting in the disruption of glycosylphosphatidylinositol biosynthesis (GPI) and therefore a deficiency of all proteins anchored to GPI in the cell membrane, such as CD55 and CD59. This produces a greater sensitivity of the complement of PNH cells, intravascular hemolysis, promotion of inflammatory mediators and systemic release of hemoglobin. In recent years, next-generation sequencing techniques have improved knowledge about the disease by changing the definition to a polygenic disease, which can define the broad clinical spectrum of the disease^2^.

Thromboembolism is the most common cause of mortality in patients with PNH. Poor survival is associated with the onset of thromboembolic complications, with a relative risk of 10.2 at 8 years^3^. There is an intrinsic relationship between coagulation and complement systems in PNH, which becomes evident when the mechanisms by which thrombosis occurs are produced. The mechanisms proposed to increase the thrombotic risk in patients with PNH are: platelet activation, complement-mediated intravascular hemolysis, alteration of the bioavailability of nitric oxide (NO), deterioration of the fibrinolytic system and release of inflammatory mediators. Although there are multiple factors that contribute to the onset of the thrombotic event, it is likely that platelet activation is the main cause of the high incidence of thrombosis associated with PNH^3^. Patients with PNH who have suffered a thrombotic event require anticoagulation^4^. Treatment with eculizumab seems to prevent the spread and recurrence of more thrombosis^5^, although this action is not complete and successful in all cases. Therefore, despite existing studies on PNH, there are still many puzzles about the mechanisms that trigger associated thrombosis, recurrence, and response to treatment with eculizumab.

During the last decade, knowledge about intercellular communication has increased in many diseases. This communication occurs through extracellular vesicles that can modulate the response of the recipient cells, which can be located remotely from the secretory cell^6^. Exosomes have been defined as small membrane vesicles, with spherical shape and diameters of 30–120 nm^7^. In the endocytosis process, the cells internalize the genetic material and the proteins in endocytic vesicles that, together with all the elements that interact with the cell membrane, will be part of the final exosome. On the other hand, the endosomes undergo a process of invagination of the membrane, accumulating intraluminal vesicles, which results in the formation of multivesicular bodies. In this process, they can mediate the uptake of proteins and genetic material from the cytoplasm of the cell^8^. Therefore, exosomes contain different components of the cell and outside the cell. The fact that the composition of the exosome is a reflection of the physiological state has confirmed that exosomes could be considered biomarkers for the diagnosis and prognosis of different diseases^9,10^. Therefore, they could participate in hematological disorders such as PNH, since it is a disease associated with thrombosis and inflammation, and is related to an increasing number of extracellular vesicles released into the plasma^11^.

Our objective here was to evaluate the differential miRNA and the proteome profile of the plasma exosomes of patients with PNH versus those of healthy subjects. This new insight could help identify surrogate biomarkers for the diagnosis and prognosis of PNH and better understand the mechanisms of underlying thrombosis in this rare disease.

## Material and Methods

### Patient and samples

Plasma samples and data from patients included in this study were provided by the National DNA Bank Carlos III (www.bancoadn.org, Salamanca, Spain) and they were processed following standard operating procedures with the appropriate approval of the Ethical and Scientific Committees. Plasma samples from patients were grouped in three different groups: (i) with no eculizumab treatment; (ii) under treatment with eculizumab that have not suffered arterial or venous thrombosis; and (iii) under treatment with eculizumab but that have suffered thrombosis. This group was named “Ecu-Thrombosis”. Group 3 was formed by patients without treatment with eculizumab. This group was named “No Ecu”. Features of each patient are described in Table 1.

**Table 1:** Demographic and clinical features of selected PNH patients.

	Group	Females/Males (F/M)	Age (years)	Treatment other than Eculizumab	Thrombosis (YES/NO)/Arterial/Venous thrombosis (AT/VE)	PNH clone in neutrophils (%)	PNH clone in monocytes (%)	LDH ratio	Direct bilirubin (mg/dL)	Total bilirubin (mg/dL)	Reticulocytes (%)	Hemoglobin (g/dL)
Patient 1	No-Ecu	M	51	Steroids	NO	92.2	96.9	8.05	0.7	3.0	<0.5	12.3
Patient 2	No-Ecu	F	13	NO	NO	39.3	43.4	2.55	0.0	1.4	4	12.7
Patient 3	No-Ecu	F	65	Anticoagulants	YES/AT	97.5	96.6	4.48	0.6	1.9	N.D.	7.7
Patient 4	Ecu-Thrombosis	M	46	NO	YES/AT	72.7	81.4	8.24	0.3	2.3	6.9	8.9
Patient 5	Ecu-Thrombosis	M	58	Anticoagulants	YES/VE	86.5	86.7	0.74	0.6	8.5	N.D.	12.3
Patient 6	Ecu-Thrombosis	M	45	NO	YES/AT	99.2	97.1	0.82	0.8	2.4	4.7	11.1
Patient 7	Ecu-no-Thrombosis	F	49	NO	NO	89.3	94.4	1.27	0.4	3.3	7.5	12.1
Patient 8	Ecu-no-Thrombosis	M	63	NO	NO	99.4	92.8	1.51	N.D.	1.5	9.5	11.9
Patient 9	Ecu-no-Thrombosis	M	64	NO	NO	97.9	100	2.62	0.8	1.7	9.2	10.1

N.D. Not Determined. LDH is represented as ratio between the actual value and the upper normal reference level.

Plasma samples from healthy subjects were provided by the Blood Transfusion Centre in Murcia (Spain) (Supplementary Table 1). None of the healthy participants had a documented history of vascular disease, a personal history of thromboembolic/hemorrhagic disease or cancer. All subjects who met eligibility criteria were enrolled after providing written informed consent. The study was approved by the Ethical Committee of our institution and performed in accordance with the ethical standards of the Declaration of Helsinki and its subsequent amendments.

Blood samples were collected by venipuncture collection and transferred to EDTA tubes. Plasma was harvested by double spun at 2500 g 15 min and frozen at −80 °C. Both patient and control plasma samples suffered a freeze/thaw cycle. Although there were oscillations in the storage time of the plasma samples, in all of them it was less than one year.

### Isolation and characterization of exosomes from plasma

Exosomes were isolated from 500 µL of plasma using Total Exosome Isolation Kit (from plasma) (Thermo Fisher Scientific, Fisher Scientific, Madrid, Spain). Final pellet was resuspended in ultrapure DEPC treated water and frozen at −80 °C. Only those exosomes that underwent the proteomic analysis, both from patients and controls, suffered a freeze/thaw cycle.

**Figure 1:**
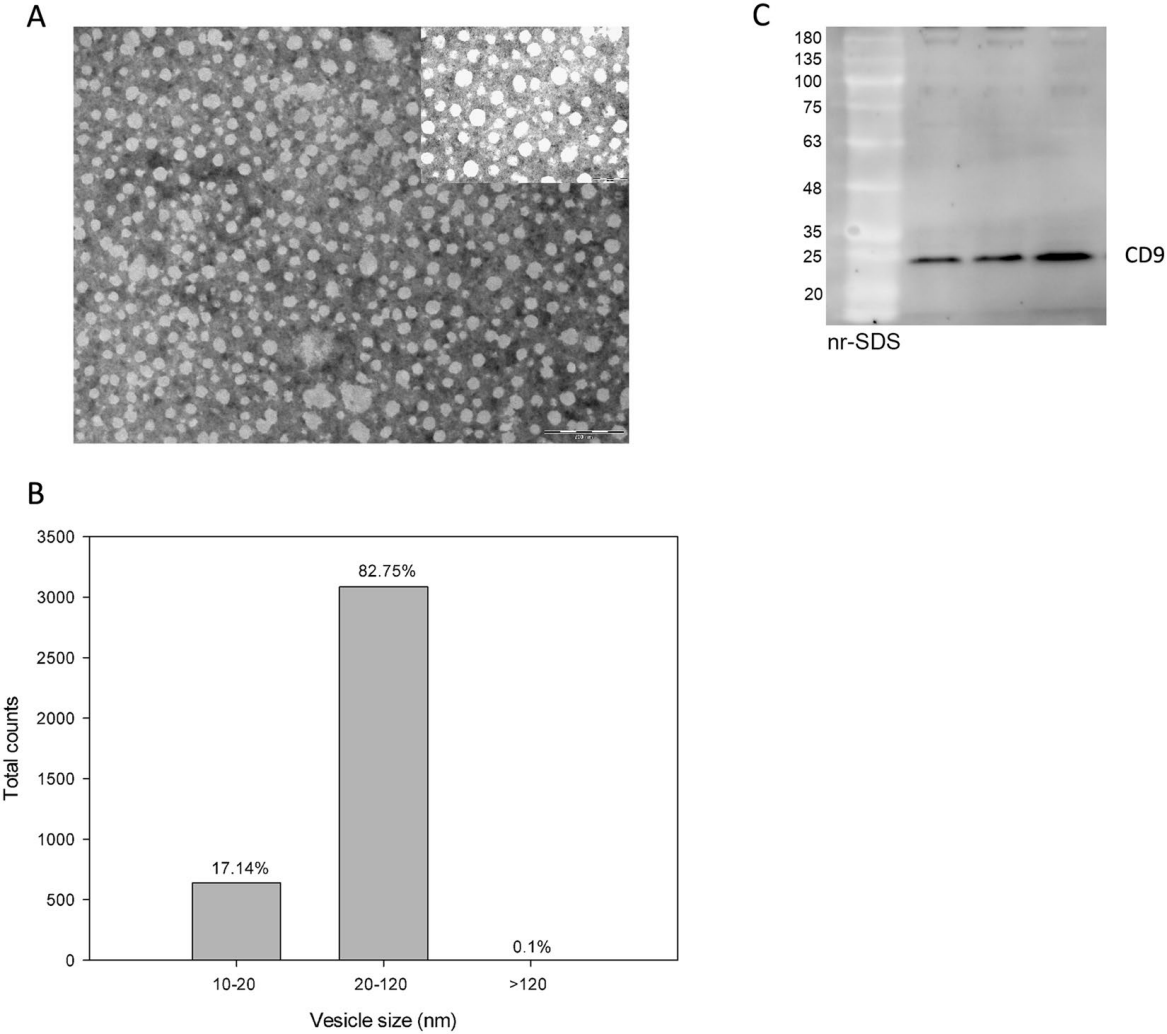
Characterization of exosomes isolated by electron microscopy and western blot. (**A**) Image of exosomes isolated at 59000 X in a Tecnai 12 transmission electron microscope with Mega-View III digital camera (Philips, Eindhoven, The Netherlands). An image acquired at 135000 X is shown in the upper right part. (**B**) Distribution by vesicle size of the isolated exosomes detected by electron microscopy. More than 100 images were recorded and the vesicles were analyzed in each one with the MIP4 Advanced software. (**C**) SDS-PAGE and western blotting of three different exosome samples isolated from plasma of control subjects. Anti-CD9 was used as primary antibody.

Exosomes isolated from the control plasma were analyzed by electron microscopy. Briefly, 20 μl of glutaraldehyde was added to 100 μl of exosomes and centrifuged at 10000 g for 5 min, 4 °C. The supernatant was discarded and the pellet was washed with H2O HyClone and centrifuged again at 10000 g 3 min, 4 °C. The pellet was resuspended in HyClonean H2O and fixed with 2% uranyl acetate for its visualization in a Tecnai 12 transmission electron microscope with Mega-View III digital camera (Philips, Eindhoven, The Netherlands). The images were acquired at 59000 X and the diameter of the isolated vesicles was analyzed with the MIP4 Advanced software. As it is shown in Fig. 1, the majority of the isolated vesicles (>80%) were in the size range between 20 and 120 nm. Moreover, SDS-PAGE and western blotting was carried out to detect CD9 as it is a common marker of exosomes.

### MiRNAome analysis

MiRNAs from exosomes were purified using Nucleo Spin miRNA Plasma Kit (Macherey-Nagel) follow the manufacturer’s protocol, incorporating to that process synthetic nucleotides UniSp2, UniSp4 and UniSp5 (internal control of isolation step). Prior addition of UniSP6 (cDNA synthesis control) on RNA, cDNA synthesis was carried out with miRCURY™ LNA™ miRNA RT Kit (Exiqon) and miRNA expression of 179 miRNA was evaluated by plasma/serum focus miRNAs PCR panel V4 (Exiqon). From 179 miRNA analyzed, we finally selected 106 miRNAs, based on the available PCR threshold cycle value (Ct) for all the samples tested. Different synthetic spike-in controls were added in the miRNA purification step (UniSp2, UniSp4 and UniSp5), retro-transcription reaction (UniSp6) and PCR (UniSp3), in order to monitoring the quality of sample processing and miRNA amplification. Data were normalized by the Ct mean value for miR-103a and miR-191-5p, which showed a high correlation with the global normalization means, resulting in a R2 = 0.9633 (p < 0.0001) and R2 = 0.9187 (p < 0.0001) for global mean and top 20 expressed miRNA geometric mean values, respectively. The expression for all 106 miRNAs previously selected was calculated using the 2−ΔCt method.”

The functional pathways analysis was studied with the mirPath web tool, setting the GO analysis as follows: GO as “GO method”, biological process as “analysis subcategory”, TargetScan as predicted algorithm, *p* < 0.01 as statistic threshold and TargetScan context score of −0.45.

### Proteomic analysis

Plasma exosomes (30 µL) were treated with trichloroacetic acid/acetone to precipitate proteins. The resulting pellets were re-dissolved with a cocktail containing 8 M urea/50 mM triethyl ammonium bicarbonate at pH 8.0 and protease inhibitors (PhosSTOP™ Roche plus cOmplete™, Mini, EDTA-free Roche). Then, aliquots containing 130 μg of protein were reduced with tris(2-carboxyethyl)phosphine for 25 min at 56 °C and alquilated with iodoacetamide. Afterwards, samples were digested overnight (pH 8.0, 37 °C) with sequencing-grade trypsin (Promega). Digestion was quenched by acidification with 1% formic acid (v/v) and peptides were desalted on C_18_ Sep-Pak columns (Waters, USA) before TMT 10-plex labelling (Thermo Fisher) following manufacturer’s instructions. To normalize study samples and the two TMT-multiplexed batches used, a pool containing all the samples was labelled with TMT-126 tag and included in each TMT batch. The different TMT 10-plex batches were desalted on C_18_ SPE before nano LC-MS analysis.

The chromatographic analysis of each TMT batch containing labelled peptides was performed in triplicate on an EASY-II nano LC equipped with a trap nano-column (Thermo Fisher, San José, CA, USA) and a C_18_ reversed phase nano-column (Nikkyo Technos Co. LTD, Japan). The chromatographic separation was performed with a continuous acetonitrile gradient using Milli-Q water (0.1% formic acid) and ACN (0.1% formic acid) as mobile phases.

Mass spectrometry analyses were performed on an LTQ-Orbitrap Velos Pro from Thermo Fisher by an enhanced FT-resolution spectrum (R = 30,000 FHMW) followed by two data dependent MS/MS scan events. One consisted of an HCD fragmentation (40% NCE) and FT-MS/MS acquisition (R = 15,000 FHMW) from the most intense ten parent ions with a charge state rejection of one and dynamic exclusion of 0.5 min. The other event consisted of a CID fragmentation (35% NCE) and IT-MS/MS acquisition from the same most intense ten parent ions which was used for peptide identification.

Protein identification/quantification was performed on Proteome Discoverer software v.1.4.0.288 (Thermo Fisher). For protein identification, all MS and MS/MS spectra were analyzed using Mascot search engine (v.2.5). The false discovery rate (FDR) and protein probabilities were calculated by Target Decoy PSM Validator working between 0.01 and 0.05 for strict and relaxed target, respectively.

For protein quantification, the ratios between each TMT-label against 126-TMT label were used and quantification results were normalized based on protein median and Log_2_ transformed for statistical analysis (Gene Spring software v. 13.1 from Agilent Technologies).

### Statistical analysis

Student’s *t*-test unpaired analyses corrected for multiple comparisons with Sidak-Bonferroni were used to select miRNA and proteins differentially expressed between PNH patients and controls. We applied *p* < 0.05 as cut-off of statistical significance and a fold-change >2.0 as cut-off of different expression levels. When we compared more than two groups Kruskal-Wallis test was used, with Benjamini & Yekutieli FDR method to control for the false discovery rate and Dunn’s test to reports the results among multiple pairwise comparisons after a Kruskal-Wallis test for stochastic dominance among groups. Statistical significance level of *p* < 0.05 was taken. To calculate the differences between patients and controls in qRT-PCR assays we used U Mann-Whitney test with *p* < 0.05 as cut-off of statistical significance. Spearman’s correlation was used to perform a lineal regression between data obtained in array experiment and qRT-PCR validation, as well as to compare miRNA expression level between exosomes and whole plasma.

## Results

### MiRNAome analysis

**Figure 2:**
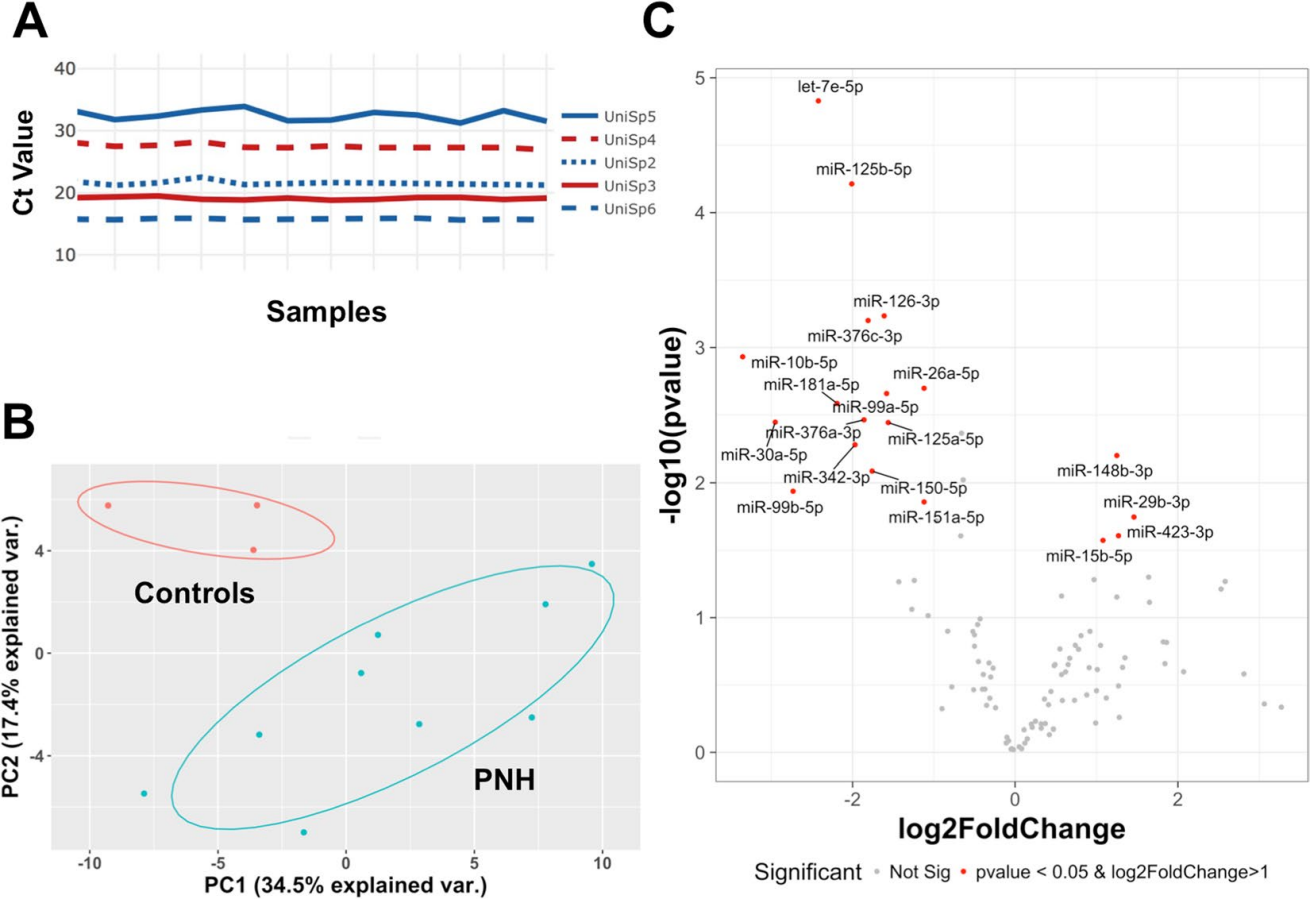
Volcano plot & box-plot graph of differentially expressed miRNAs in PNH patients. (**A**) Volcano plot represented all miRNA fold (log2fold) and *p*-value [−log10 (*p*-value)] values. The miRNAs pointed are those that met the criteria of significance (*p*-value < 0.05 and fold change in log2 > 1, represented by horizontal dashed line and vertical dashed lines, respectively). Box-plot showed median, interquartile range contained middle 50% values, minimum and maximum values, excluding outliers and kernel probability density. (**B**) MiRNAs up-regulated in PNH patients. (**C**) MiRNAs down-regulated in PNH patients (only the 4 miRNAs with lowest *p*-value).

Similar Ct values were observed across the 12 samples for all spike-in controls (Supplementary Fig. 1A). We identified a differential expression profile between controls and patients with PNH by principal component analysis (Supplementary Fig. 1B). Volcano plot analysis (Fig. 2A) identified four miRNAs (miR-148b-3p, miR29b-3p, miR-423-3p, and miR-15b-5p) over-expressed at least 2-fold in PNH patients in comparison with control samples (Fig. 2B). Alternatively, we identified 15 miRNAs (let-7e-5p, miR-125b-5p, miR-126-3p, miR-376c-3p, miR-10b-5p, miR-26a-5p, miR-181a-5p, miR99a-5p, miR-376a-3p, miR-125a-5p, miR30a-5p, miR-342-3p, miR-150-5p, miR-99b-5p, and miR-151a-5p) under-expressed at least 2-fold in PNH patients in comparison with controls (Fig. 2A,C) only represented the four miRNAs with the lowest *p*-value.

**Figure 3:**
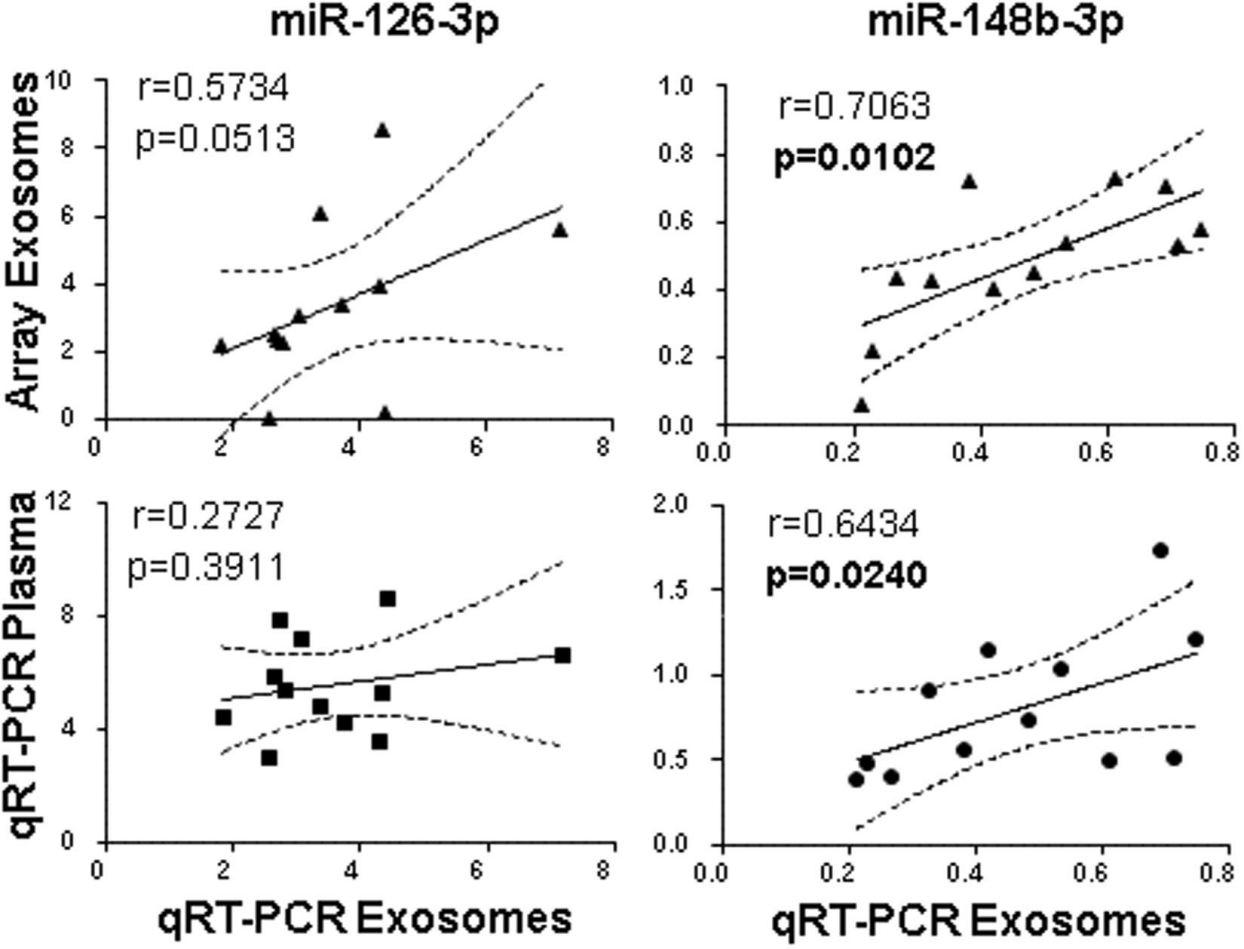
Correlation of array exosomes PCR between qRT-PCR in exosomes and qRT-PCR in total plasma. Pearson correlation showed lineal regression (black line) and 95% confidence interval (dashed lines). Each graph shows Pearson’s correlation coefficient (r) and *p*-value (*p*) in bold if the value is below to 0.05. Correlation of one miRNA down-regulated in PNH patients (miR-126-3p) and one miRNA up-regulated in PNH patients (miR-148b-3p).

In order to test if the same expression pattern was obtained in plasma, we selected the two miRNAs, miR-148b-3p and miR-126-3p, with the higher differential expression between PNH patients and controls. As shown in Fig. 3 (upper panels) both miRNAs were technically validated in exosomes. However, we observed a different miR-126-3p profile between exosomes and plasma. For miR-148b-3p, the plasma profile was similar to that found in exosomes (r = 0.6434, *p* = 0.0240) (Fig. 3A, lower panels).

We next investigated potential targets of the differentially expressed miRNAs (controls vs. PNH patients) by performing an *in silico* study, in order to find biological pathways that may be altered in PNH patients. Supplementary Table 2 showed a list of biological pathways sorted by *p* < 0.001.

### Proteomic analysis

In total, 145 different proteins were identified, although only 132 were quantified. Supplementary Fig. 2 shows the PCA and hierarchical clustering analyses for those proteins differentially found in PNH patients *vs* healthy controls. According to these results, 18 identified proteins could be involved in PNH, including an increase of 4.9 and −2.0-fold in hemoglobin and haptoglobin levels, respectively, in patients in comparison with controls. It is also noteworthy the 1.5-fold decrease (*p* = 0.005) of Protein S in PNH patients *versus* controls, a trend that was also followed by the C4BP protein (Supplementary Table 3). Differential proteomic analysis did not reveal significant differences when comparing the three groups of patients, except for the 3.9-fold increase of Ig heavy chain V-I region HG3 levels in eculizumab-treated patients in comparison with those without treatment (*p* < 0.001) (Supplementary Table 4). Finally, from the proteins identified, those proteins differentially expressed in patients and controls were grouped taking into account the pathways in which they are present. As it is shown in Supplementary Table 5, these pathways were mainly related to the complement system and the immune response.

## Discussion

PNH is a rare disease in which thrombosis is the main leading cause of death^3^. The risk of thrombosis in patients with PNH appears to be multifactorial.

We present a descriptive study of patients with classic PNH that provides information on the content of circulating plasma exosomes and describes the differences between patients in different clinical situations. Our results suggest that exosomes may reflect the patient’s condition and may provide key information about predisposition to thrombosis. Unfortunately, there are many limitations in this type of study, since patients with PNH are not homogeneous and, for example, those patients who have not suffered thrombosis at the time of blood collection could develop a thrombotic event in the future.

To our knowledge, there are no previous reports on the plasma expression of miRNA in patients with PNH. In the present study, we found differential expression of several miRNAs in plasma exosomes between controls and patients with PNH.

Blood miRNAs have also been associated with pathophysiology and there are several studies that characterize miRNAs circulating in plasma^12,13^. Knowing that miRNAs can be found in different protein complexes, such as the Ago2-miRNA complex, which circulates freely or as part of exosomes, our approach was to measure the expression of miRNAs in exosomes. The use of whole plasma is easier and allows future standardizations. However, not all miRNAs examined in whole plasma showed the same profile observed in exosomes. We studied the two miRNAs differentially expressed in exosomes between the controls and the patients with PNH, and we only found miR-148b-3p, showing a strong correlation between the total plasma fraction and the exosome content, which could significantly increase the applicability of our findings.

Next, we evaluated the potential targets of differentially expressed miRNAs and none of the miRNAs that are expressed differently between patients and controls regulate the expression of proteins detected in exosomes. This could be explained by the fact that these miRNAs regulate the expression of certain genes in the recipient cells where the exosome is captured^14^. We then carried out an *in silico* evaluation of the theoretical objectives of miRNAs differentially expressed in plasma exosomes of patients with PNH, and we found that 18 pathways can be regulated (P < 0.001), i.e., “biosynthetic process”, “cell signaling” -cellular “,” Small molecule metabolic process “,” biological process “or” catabolic process “(Supplementary Table 2). Although this type of analysis has its limitations in our study, such as, that we do not know the origin of the miRNA found in circulating peripheral blood exosomes; or that a miRNA can be target hundreds of transcripts, we found interesting pathways related to protein modification, i.e., “cellular protein modification”, “post-translational protein modification”, “phosphatidylinositol-mediated signaling” and “glycosaminoglycan metabolic process”, which may explain the functional differences between patients with PNH and healthy controls beyond the known mutations in PIGA^15^.

Concerning the proteins differently detected between the exosomes of the patients and the controls, the hemoglobin showed the highest score. The level of free hemoglobin, and in consequence of haptoglobin, is a characteristic of patients with PNH and contributes to the pro-thrombotic state with the consequent consumption of NO, fibrinolytic defects and the proinflammatory effects of C5a^16^. However, the effect of this hemoglobin on the recipient cells is unknown. Proteomic analysis also showed reduced levels of protein S and C4 binding proteins in patients with PNH compared to healthy subjects. Protein S functions as a cofactor of protein C in the inactivation of factor Va (FVa) and FVIIIa^17^. Protein S also binds to the complement complex C5,6,7 and prevents the insertion into the membrane. This function prevents the inappropriate activation of the complement system, which would cause uncontrolled systemic inflammation. Currently, we do not know if the protein S and the C4 binding protein exist as a complex in plasma exosomes, but the dysregulation of coagulation and inflammation in these patients suggests that the decrease in protein S levels could contribute to the alteration of both systems.

Analysis of the pathways affected by differential protein levels found in PNH versus healthy subjects revealed that 8 pathways were significantly involved (Supplementary Table 5). Among them, those dealing with platelets should be highlighted, such as the interactions of the cell surface in the vascular wall and the binding of the GPCR ligand and downstream signaling. It is also noteworthy that there are other pathways, not statistically significant in the analysis, but closely related to PNH or thrombosis, such as the complement and the coagulation cascade, the effects of nitric oxide, platelet aggregation, the formation of blood clots, fibrin, etc.

We are aware of the low number of patients participating in the present study. PNH is a rare disease with extensive clinical expression; this justifies the great difficulty of studying a homogeneous group. In addition, one of the main questions derived from our study is whether the differential expression of proteins and miRNAs in the plasma exosomes of patients with PNH is a cause or consequence of the pathological state of the patients. This cannot be answered, but our results could help to understand the patient’s situation at the time of the blood draw. In summary, we have described here for the first time the content in miRNA and proteins in plasma exosomes from patients with PNH. Differentially expressed miRNA and proteins are mainly associated with the complement system and the inflammatory response, which may influence the pathological status of patients with PNH. While the interaction between inflammation and thrombosis is well-known^18^, which may be a common feature in many patients with PNH, more research is needed with larger cohorts to confirm whether some of the proteins and miRNAs studied here could be useful as surrogate biomarkers of PNH.

## Supplementary information

Supplementary Information

## Data Availability

Raw and normalized qRT-PCR data has been uploaded to Gene Expression Omnibus public database (GEO) with accession number #GSE122352. The mass spectrometry proteomics data have been deposited to the ProteomeXchange Consortium via the PRIDE^19,20^ partner repository with the dataset identifier PXD011768.
