# Supplementary material for: Differential miRNA expression profile and proteome in plasma exosomes from patients with paroxysmal nocturnal hemoglobinuria
Source: Sci Rep. 2019 Mar 5;9:3611. doi: 10.1038/s41598-019-40453-5 (PMC6401143; doi:10.1038/s41598-019-40453-5)

Differential miRNA expression profile and proteome in plasma exosomes from patients with paroxysmal nocturnal hemoglobinuria

Raúl Teruel-Montoya^1,2*^, Ginés Luengo-Gil^1^, Fernando Vallejo^3^, José Enrique Yuste^3^, Nataliya Bohdan^1^, Nuria García-Barberá^1^, Salvador Espín^1^, Constantino Martínez^1^, Juan Carlos Espín^4^, Vicente Vicente^1,2^, Irene Martínez-Martínez^1,2*^

^1^Servicio de Hematología y Oncología Médica, Hospital Universitario Morales Meseguer, Centro Regional de Hemodonación, Universidad de Murcia, IMIB-Arrixaca, Murcia.

^2^Grupo de Investigación CB15/00055, Centro de Investigación Biomédica en Red de Enfermedades Raras (CIBERER), Instituto de Salud Carlos III (ISCIII).

^3^Servicio de Metabolómica, CEBAS-CSIC, 30100 Campus de Espinardo, Murcia (Spain).

^4^Laboratory of Food & Health, Group of Quality, Safety and Bioactivity of Plant Foods, CEBAS-CSIC, 30100 Campus de Espinardo, Murcia (Spain).

* **Corresponding authors’ contact information**:

Dr. Irene Martínez-Martínez. E-mail: [immlgi@um.es](mailto:immlgi@um.es)

Dr. Raúl Teruel-Montoya. E-mail: [raulteruelmontoya@hotmail.com](mailto:vicente.vicente@carm.es)

Centro Regional de Hemodonación

Ronda de Garay S/N, Murcia 30003, Spain

Tel: +34968341990

Fax: +34968261914

SUPPLEMENTARY MATERIAL

**
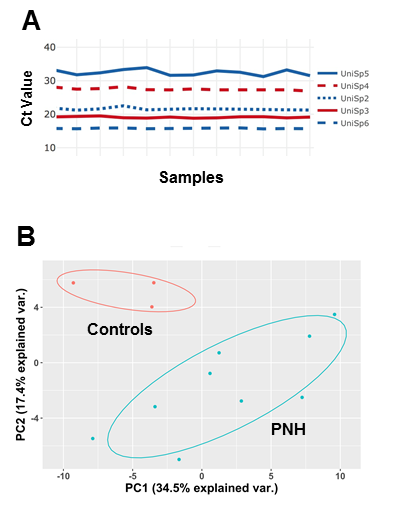
**

**Supplementary Figure 1.** **Quality spike-in controls & Principal Component Analysis (PCA) of the results obtained from the miRNA expression profile study.** A) Representation across samples of Ct value of different synthetic controls templates. UniSp2, UniSp4 and UniSp5 spike-in mix are used as RNA isolation controls, UniSp6 as cDNA synthesis RNA control and UniSp2 as PCR amplification control. B) PCA analysis showed two clearly differentiated groups.

**
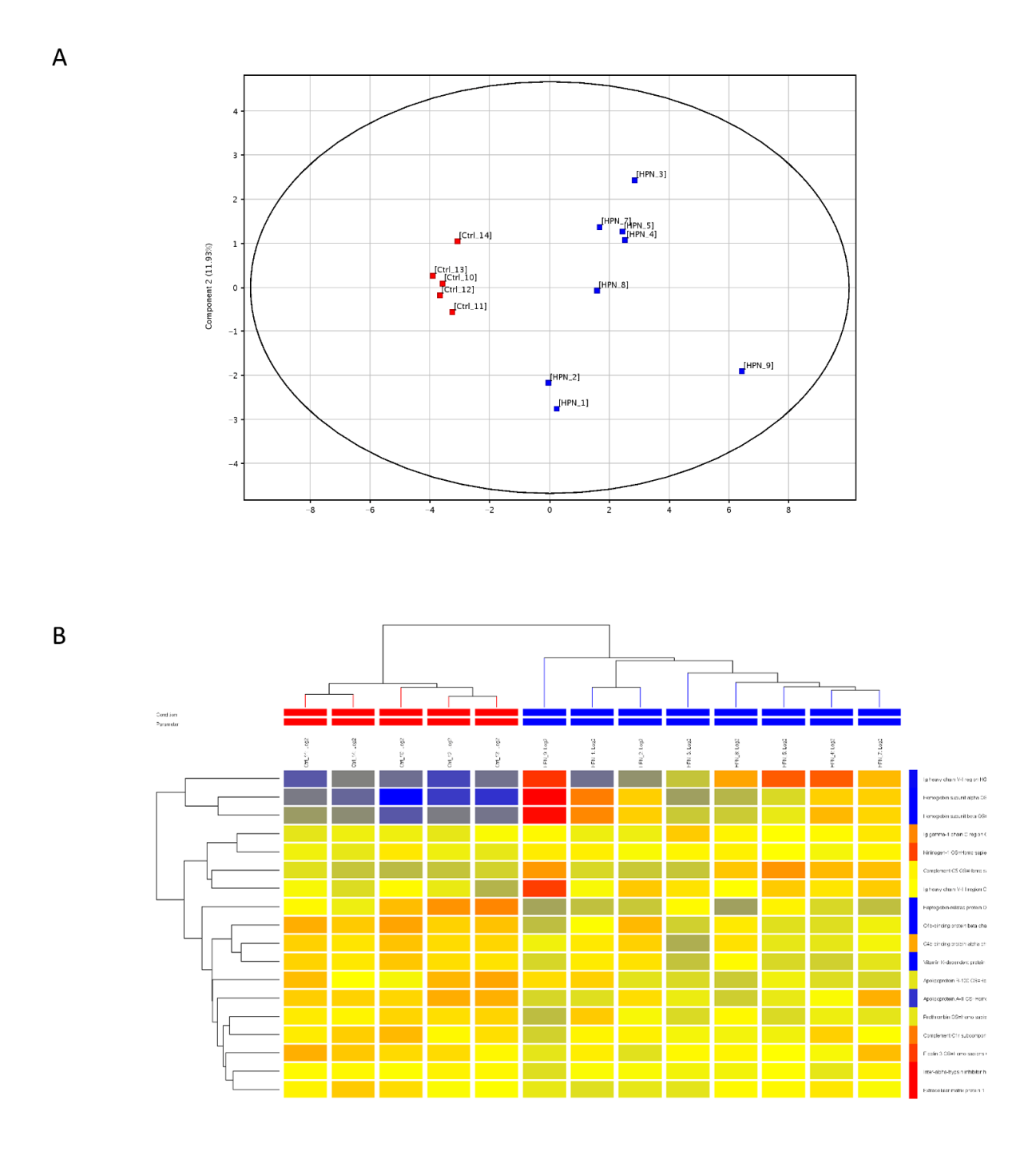
**

**Supplementary Figure 2. Principal Component Analysis (PCA) and Hierarchical clustering analysis of the results obtained from the proteomic study**. A) PCA of samples. Patient 6 described in Table 1 was excluded because this sample was outside of the 95% confidence region. B) Hierarchical clustering analysis. Cluster diagram with identified proteins along the vertical axis and cases and controls orientated along the horizontal axis.

**Supplementary Table 1. Features of control subjects**

| Subject | Gender | Age |
| --- | --- | --- |
| 1 | Male | 23 |
| 2 | Male | 44 |
| 3 | Male | 30 |
| 4 | Female | 58 |
| 5 | Female | 27 |

**Supplementary Table 2.** MiRNA pathway analysis using DIANA-mirPath. The significant biological processes are sorted by p-value.

| **GO Category** | **p-value** | **#genes** | **#miRNAs** |
| --- | --- | --- | --- |
| Cellular nitrogen compound metabolic process (GO:0034641) | 3,53E-11 | 261 | 19 |
| Collagen catabolic process (GO:0030574) | 3,64E-09 | 19 | 6 |
| Extracellular matrix disassembly (GO:0022617) | 7,86E-09 | 20 | 6 |
| Neurotrophin TRK receptor signaling pathway (GO:0048011) | 8,06E-09 | 28 | 11 |
| Biosynthetic process (GO:0009058) | 3,23E-08 | 221 | 19 |
| Cellular protein modification process (GO:0006464) | 4,61E-07 | 138 | 19 |
| Extracellular matrix organization (GO:0030198) | 2,23E-06 | 38 | 9 |
| Fc-epsilon receptor signaling pathway (GO:0038095) | 2,06E-05 | 17 | 9 |
| Fibroblast growth factor receptor signaling pathway (GO:0008543) | 3,99E-05 | 22 | 12 |
| Epidermal growth factor receptor signaling pathway (GO:0007173) | 5,28E-05 | 22 | 9 |
| Cell-cell signaling (GO:0007267) | 3,78E-04 | 48 | 15 |
| Post-translational protein modification (GO:0043687) | 5,88E-04 | 16 | 12 |
| Phosphatidylinositol-mediated signaling (GO:0048015) | 7,35E-04 | 16 | 10 |
| Small molecule metabolic process (GO:0044281) | 2,42E-03 | 121 | 19 |
| Biological_process (GO:0008150) | 2,54E-03 | 840 | 19 |
| Catabolic process (GO:0009056) | 3,72E-03 | 104 | 17 |
| Glycosaminoglycan metabolic process (GO:0030203) | 9,40E-03 | 11 | 8 |
| Blood coagulation (GO:0007596) | 9,40E-03 | 29 | 13 |

**Supplementary Table 3. Proteins contained in exosomes from patients with PNH and controls that resulted statistically significant using T-test.** Significant proteins were considered when p<0.05. Fold Change is also indicated. CTRL: control.

| Swiss-Prot ID | Protein Name | p-value | Regulation | Fold Change (PNH/CTRL) |
| --- | --- | --- | --- | --- |
| P69905 | Hemoglobin subunit alpha | 8,1E-04 | up | 4,9 |
| P68871 | Hemoglobin subunit beta | 1,2E-03 | up | 3,4 |
| P00739 | Haptoglobin-related protein | 3,1E-03 | down | -2,0 |
| P20851 | C4b-binding protein beta chain | 3,4E-03 | down | -1,7 |
| P07225 | Vitamin K-dependent protein S | 5,5E-03 | down | -1,5 |
| P01743 | Ig heavy chain V-I region HG3 | 6,2E-03 | up | 3,9 |
| P02652 | Apolipoprotein A-II | 9,0E-03 | down | -1,5 |
| P04114 | Apolipoprotein B-100 | 2,1E-02 | down | -1,5 |
| P00734 | Prothrombin | 2,1E-02 | down | -1,4 |
| P01768 | Ig heavy chain V-III region CAM | 2,3E-02 | up | 1,6 |
| P01031 | Complement C5 | 2,4E-02 | up | 1,7 |
| P04003 | C4b-binding protein alpha chain | 3,0E-02 | down | -1,4 |
| P01857 | Ig gamma-1 chain C region | 3,2E-02 | up | 1,2 |
| P00736 | Complement C1r subcomponent | 3,3E-02 | down | -1,3 |
| P01042 | Kininogen-1 | 3,8E-02 | up | 1,1 |
| O75636 | Ficolin-3 | 3,8E-02 | down | -1,3 |
| Q14624 | Inter-alpha-trypsin inhibitor heavy chain H4 | 4,7E-02 | down | -1,1 |
| Q16610 | Extracellular matrix protein 1 | 4,7E-02 | down | -1,2 |

**Supplementary Table 4. Proteins contained in exosomes from patients with PNH and controls that resulted statistically significant using ANOVA test among groups.** Significant proteins were considered when p<0.05. Fold Change (FC) is also indicated. CTRL: control.

| **Swiss-Prot ID** | **Protein Name** | **p-value** | **FC ([No-Ecu] vs [CTRL])** | **Regulation ([No-Ecu] vs [CTRL])** | **FC ([Ecu-No Throm] vs [CTRL])** | **Regulation ([Ecu-No Throm] vs [CTRL])** | **FC ([Ecu-Throm] vs [CTRL])** | **Regulation ([Ecu-Throm] vs [CTRL])** | **FC ([Ecu-No Throm] vs [No-Ecu])** | **Regulation ([Ecu-No Throm] vs [No-Ecu])** | **FC ([Ecu-Throm] vs [No-Ecu])** | **Regulation ([Ecu-Throm] vs [No-Ecu])** | **FC ([Ecu-Throm] vs [Ecu-No throm])** | **Regulation ([Ecu-Throm] vs [Ecu-No throm])** |
| --- | --- | --- | --- | --- | --- | --- | --- | --- | --- | --- | --- | --- | --- | --- |
| P01743 | Ig heavy chain V-I region HG3 | 4,68E-05 | 1,44 | up | **5,93** | up | **6,29** | up | **4,12** | up | **4,37** | up | 1,06 | up |
| P01861 | Ig gamma-4 chain C region | 8,46E-04 | **-2,53** | down | **2,20** | up | 1,45 | up | **5,57** | up | **3,68** | up | -1,51 | down |
| P01024 | Complement C3 | 1,61E-03 | **1,26** | up | 1,19 | up | -1,09 | down | -1,06 | down | **-1,38** | down | **-1,30** | down |
| P10643 | Complement component C7 | 6,15E-03 | -1,06 | down | **2,12** | up | 1,60 | up | **2,24** | up | 1,69 | up | -1,32 | down |
| P00739 | Haptoglobin-related protein | 6,88E-03 | -1,84 | down | -1,66 | down | **-2,56** | down | 1,11 | up | -1,39 | down | -1,54 | down |
| P07225 | Vitamin K-dependent protein S | 8,39E-03 | -1,31 | down | -1,77 | down | -1,46 | down | -1,36 | down | -1,12 | down | 1,22 | up |
| P69905 | Hemoglobin subunit alpha | 9,49E-03 | **4,52** | up | 3,56 | up | **6,11** | up | -1,27 | down | 1,35 | up | 1,71 | up |
| P01031 | Complement C5 | 1,02E-02 | 1,02 | up | 1,73 | up | **2,27** | up | 1,70 | up | **2,23** | up | 1,31 | up |
| P20851 | C4b-binding protein beta chain | 1,14E-02 | -1,45 | down | -2,20 | down | -1,73 | down | -1,51 | down | -1,19 | down | 1,27 | up |
| Q16610 | Extracellular matrix protein 1 | 1,38E-02 | **-1,37** | down | **-1,03** | down | -1,18 | down | **1,32** | up | 1,16 | up | -1,14 | down |
| P68871 | Hemoglobin subunit beta | 1,49E-02 | 3,20 | up | 2,63 | up | **4,04** | up | -1,21 | down | 1,26 | up | 1,53 | up |
| P02790 | Hemopexin | 3,61E-02 | **-2,22** | down | -1,29 | down | -1,11 | down | 1,73 | up | 2,00 | up | 1,16 | up |
| P01780 | Ig heavy chain V-III region JON | 4,90E-02 | 1,07 | up | 1,38 | up | 1,78 | up | 1,29 | up | 1,65 | up | 1,28 | up |

**Supplementary Table 5. Pathway list generated from proteins statically significant identified in exosomes from patients with PNH.** Pathways represented in bold are those statistically significant in “Pathways Analysis” on GeneSpring Pathway Architect software (v.13.1)

| Pathway | p-value | Matched Proteins | Pathway Proteins |
| --- | --- | --- | --- |
| **Cell surface interactions at the vascular wall** | **6,0E-03** | **3** | **91** |
| **Selenium Micronutrient Network** | **1,4E-02** | **4** | **84** |
| **PTM-gamma carboxylation, hypusine formation and arylsulfatase activation** | **1,5E-02** | **2** | **15** |
| **GPCR downstream signaling** | **2,6E-02** | **3** | **406** |
| **GPCR ligand binding** | **2,6E-02** | **3** | **371** |
| **Gastrin-CREB signaling pathway via PKC and MAPK** | **4,1E-02** | **2** | **147** |
| **Uptake of Carbon Dioxide and Release of Oxygen by Erythrocytes** | **4,1E-02** | **2** | **8** |
| **Uptake of Oxygen and Release of Carbon Dioxide by Erythrocytes** | **4,1E-02** | **2** | **8** |
| Complement and Coagulation Cascades | 1,1E-01 | 5 | 64 |
| Angiogenesis overview | 1,2E-01 | 1 | 65 |
| Effects of Nitric Oxide | 1,2E-01 | 1 | 8 |
| Factors involved in megakaryocyte development and platelet production | 1,2E-01 | 1 | 71 |
| IL1 and megakaryotyces in obesity | 1,2E-01 | 1 | 24 |
| Platelet Aggregation (Plug Formation) | 1,2E-01 | 1 | 10 |
| Platelet homeostasis | 1,2E-01 | 1 | 31 |
| Thrombin signalling through proteinase activated receptors (PARs) | 1,2E-01 | 1 | 15 |
| Formation of Fibrin Clot (Clotting Cascade) | 1,4E-01 | 3 | 30 |
| ACE Inhibitor Pathway | 2,3E-01 | 1 | 17 |
| Folate Metabolism | 2,3E-01 | 1 | 67 |
| Regulation of Insulin-like Growth Factor (IGF) Transport and Uptake by Insulin-like Growth Factor Binding Proteins (IGFBPs) | 2,3E-01 | 1 | 13 |
| Spinal Cord Injury | 2,3E-01 | 1 | 116 |
| Statin Pathway | 2,6E-01 | 2 | 31 |
| Visual phototransduction | 3,1E-01 | 2 | 67 |
| Allograft Rejection | 3,3E-01 | 1 | 100 |
| PPAR alpha pathway | 3,3E-01 | 1 | 26 |
| Regulation of Actin Cytoskeleton | 3,3E-01 | 1 | 148 |
| Regulation of Lipid Metabolism by Peroxisome proliferator-activated receptor alpha (PPARalpha) | 3,3E-01 | 1 | 118 |
| Vitamin B12 Metabolism | 3,3E-01 | 1 | 53 |
| Human Complement System | 3,3E-01 | 5 | 136 |
| Lipid digestion, mobilization, and transport | 3,6E-01 | 2 | 41 |
| Nuclear Receptors Meta-Pathway | 4,2E-01 | 1 | 318 |
| Complement Activation, Classical Pathway | 5,0E-01 | 2 | 17 |
| Complement cascade | 6,4E-01 | 9 | 192 |
| Blood Clotting Cascade | 6,6E-01 | 1 | 22 |
| Binding and Uptake of Ligands by Scavenger Receptors | 7,9E-01 | 6 | 195 |
| Fcgamma receptor (FCGR) dependent phagocytosis | 9,8E-01 | 3 | 210 |
| Fc epsilon receptor (FCERI) | 9,9E-01 | 2 | 218 |
| Immunoregulatory interactions between a Lymphoid and a non-Lymphoid cell | 9,9E-01 | 2 | 292 |
| Signaling by the B Cell Receptor (BCR) | 9,9E-01 | 2 | 239 |

**Full-length blot of different exosome samples isolated from plasma of control subjects**. Anti-CD9 was used as primary antibody. The area displayed with a rectangle was used in Figure 1C.


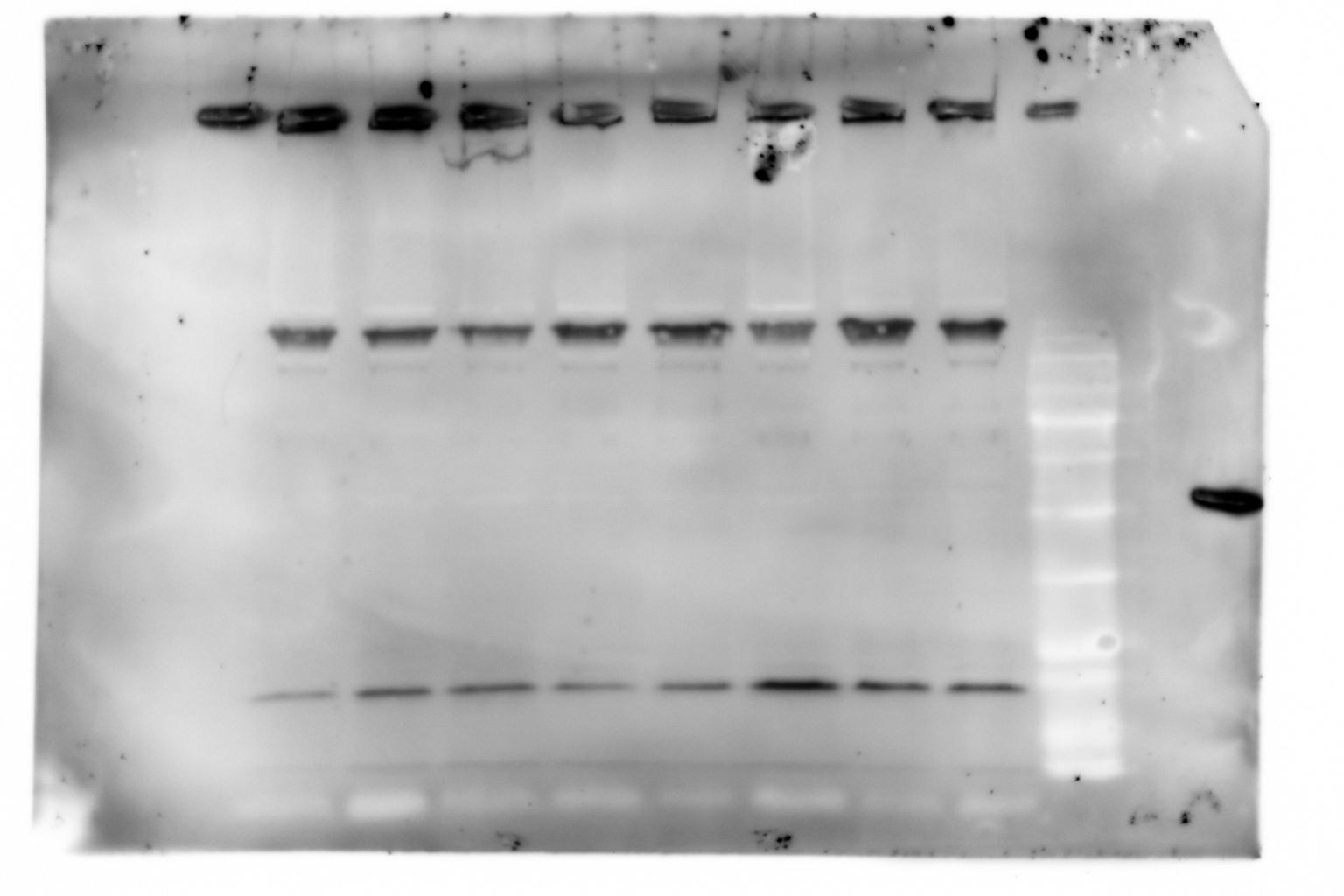

Supplement: Supplementary file 1 — Supplementary Information [file 41598_2019_40453_MOESM1_ESM.docx]
